# 2-Bromo-*N*-(2-chloro­phen­yl)acetamide

**DOI:** 10.1107/S1600536809028918

**Published:** 2009-07-25

**Authors:** B. Thimme Gowda, Sabine Foro, P. A. Suchetan, Hartmut Fuess, Hiromitsu Terao

**Affiliations:** a Department of Chemistry, Mangalore University, Mangalagangotri 574 199, Mangalore, India; b Institute of Materials Science, Darmstadt University of Technology, Petersenstrasse 23, D-64287 Darmstadt, Germany; c Faculty of Integrated Arts and Sciences, Tokushima University, Minamijosanjima-cho, Tokushima 770-8502, Japan

## Abstract

The conformation of the N—H bond in the structure of the title compound, C_8_H_7_BrClNO, is *syn* to the 2-chloro substituent in the aniline ring and *anti* to both the C=O and C—Br bonds in the side chain, similar to that observed in 2-chloro-*N*-(2-chloro­phen­yl)acetamide. In the crystal, mol­ecules are linked into chains along the *a* axis by N—H⋯O hydrogen bonds. These chains are in turn linked into pairs, in the form of columns, through much weaker C—H⋯Cl and Br⋯Br [4.3027 (3) Å] inter­actions.

## Related literature

For the preparation of the compound, see: Gowda *et al.* (2003 ▶). For our studies of the effect of ring and side-chain substituents on the structures of *N*-aromatic amides, see: Gowda *et al.* (2007*a* ▶,*b* ▶,*c* ▶)
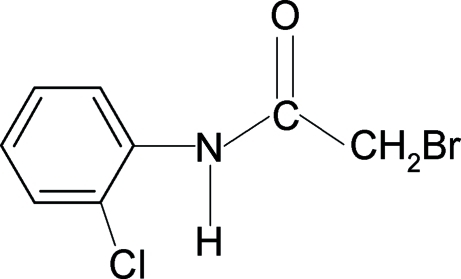


## Experimental

### 

#### Crystal data

C_8_H_7_BrClNO
*M* *_r_* = 248.51
Monoclinic, 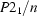

*a* = 9.9781 (9) Å
*b* = 4.7161 (5) Å
*c* = 20.028 (2) Å
β = 102.194 (9)°
*V* = 921.21 (16) Å^3^
*Z* = 4
Cu *K*α radiation
μ = 8.36 mm^−1^
*T* = 299 K
0.55 × 0.20 × 0.15 mm

#### Data collection

Enraf–Nonius CAD-4 diffractometer
Absorption correction: ψ scan (North *et al.*, 1968 ▶) *T*_min_ = 0.071, *T*_max_ = 0.286
2349 measured reflections
1650 independent reflections
1482 reflections with *I* > 2σ(*I*)
*R*_int_ = 0.023
3 standard reflections frequency: 120 min intensity decay: 1.0%

#### Refinement

*R*[*F* ^2^ > 2σ(*F* ^2^)] = 0.044
*wR*(*F* ^2^) = 0.118
*S* = 1.11
1650 reflections
113 parameters
1 restraint
H atoms treated by a mixture of independent and constrained refinement
Δρ_max_ = 0.87 e Å^−3^
Δρ_min_ = −0.88 e Å^−3^

### 

Data collection: *CAD-4-PC* (Enraf–Nonius, 1996 ▶); cell refinement: *CAD-4-PC*; data reduction: *REDU4* (Stoe & Cie, 1987 ▶); program(s) used to solve structure: *SHELXS97* (Sheldrick, 2008 ▶); program(s) used to refine structure: *SHELXL97* (Sheldrick, 2008 ▶); molecular graphics: *PLATON* (Spek, 2009 ▶); software used to prepare material for publication: *SHELXL97*.

## Supplementary Material

Crystal structure: contains datablocks I, global. DOI: 10.1107/S1600536809028918/bg2279sup1.cif

Structure factors: contains datablocks I. DOI: 10.1107/S1600536809028918/bg2279Isup2.hkl

Additional supplementary materials:  crystallographic information; 3D view; checkCIF report

## Figures and Tables

**Table 1:** Hydrogen-bond geometry (Å, °)

*D*—H⋯*A*	*D*—H	H⋯*A*	*D*⋯*A*	*D*—H⋯*A*
N1—H1*N*⋯O1^i^	0.839 (19)	2.05 (2)	2.852 (4)	160 (4)
C8—H8*B*⋯Cl1^ii^	0.97	3.09	3.765 (5)	128

Symmetry codes: (i) 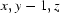; (ii) 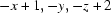.

**Fig. 1.**
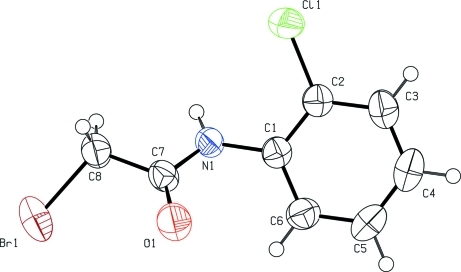
Molecular structure of (I), showing the atom labelling scheme. Displacement ellipsoids are drawn at the 50% probability level and H atoms are represented as small spheres of arbitrary radii.

**Fig. 2.**
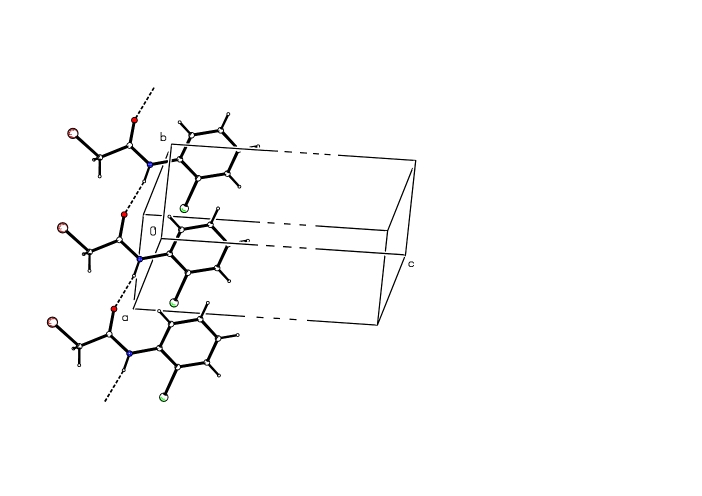
Molecular packing of (I) showing chains, with hydrogen bonds shown as dashed lines. The unit cell is shown as "broken" along the c direction, for completeness.

## supplementary crystallographic information

### Comment

As part of a study of the effect of the ring and the side chain substituents on the structures of N-aromatic amides (Gowda *et al.*, 2007*a*,*b*,*c*), in the present work we report the structure of 2-bromo-*N*-(2-chlorophenyl)acetamide (I). The conformation of the N—H bond in the structure is *syn* to the *ortho*-Cl substituent in the aniline ring and *anti* to both the C=O and C—Br bonds in the side chain (Fig. 1), similar to that observed in 2-chloro-*N*-(2-chlorophenyl)acetamide (Gowda *et al.*, 2007*a*) and other side chain substituted aromatic amides.

The packing diagram in Fig. 2 shows the formation of molecular chains in the direction of the *a* axis through the N1—H1N···O1 H-bonds (Table 1). These chains are in turn linked into pairs, in the form of strips, through much weaker C—H···Cl and Br···Br interactions.

### Experimental

The title compound was prepared from 2-chloroaniline and bromoacetylchloride according to the literature method (Gowda *et al.*, 2003). The purity of the compound was checked by determining its melting point, and further characterized by recording its infrared spectra (Gowda *et al.*, 2003). Single crystals of the title compound used for X-ray diffraction studies were obtained by slow evaporation of an ethanolic solution at room temperature.

### Refinement

The N-bound H atom was located in a difference map and refined with a restrained geometry (N-H= 0.86 (2) Å). The other H atoms were positioned with idealized geometry using a riding model [C—H = 0.93–0.97 Å]. All H atoms were refined with isotropic displacement parameters (set to 1.2 times of the *U*_eq_ of the parent atom).

### Crystal data

**Table:**

C_8_H_7_BrClNO	*F*(000) = 488
*M**_r_* = 248.51	*D*_x_ = 1.792 Mg m^−^^3^
Monoclinic, *P*2_1_/*n*	Cu *K*α radiation, λ = 1.54180 Å
Hall symbol: -P 2yn	Cell parameters from 25 reflections
*a* = 9.9781 (9) Å	θ = 7.3–22.5°
*b* = 4.7161 (5) Å	µ = 8.36 mm^−^^1^
*c* = 20.028 (2) Å	*T* = 299 K
β = 102.194 (9)°	Rod, colourless
*V* = 921.21 (16) Å^3^	0.55 × 0.20 × 0.15 mm
*Z* = 4	

### Data collection

**Table:**

Enraf–Nonius CAD-4 diffractometer	1482 reflections with *I* > 2σ(*I*)
Radiation source: fine-focus sealed tube	*R*_int_ = 0.023
graphite	θ_max_ = 67.0°, θ_min_ = 4.5°
ω/2θ scans	*h* = −11→3
Absorption correction: ψ scan (North *et al.*, 1968)	*k* = −5→0
*T*_min_ = 0.071, *T*_max_ = 0.286	*l* = −23→23
2349 measured reflections	3 standard reflections every 120 min
1650 independent reflections	intensity decay: 1.0%

### Refinement

**Table:**

Refinement on *F*^2^	Secondary atom site location: difference Fourier map
Least-squares matrix: full	Hydrogen site location: inferred from neighbouring sites
*R*[*F*^2^ > 2σ(*F*^2^)] = 0.044	H atoms treated by a mixture of independent and constrained refinement
*wR*(*F*^2^) = 0.118	*w* = 1/[σ^2^(*F*_o_^2^) + (0.0557*P*)^2^ + 1.2453*P*] where *P* = (*F*_o_^2^ + 2*F*_c_^2^)/3
*S* = 1.11	(Δ/σ)_max_ = 0.002
1650 reflections	Δρ_max_ = 0.87 e Å^−^^3^
113 parameters	Δρ_min_ = −0.88 e Å^−^^3^
1 restraint	Extinction correction: *SHELXL97* (Sheldrick, 2008), Fc^*^=kFc[1+0.001xFc^2^λ^3^/sin(2θ)]^-1/4^
Primary atom site location: structure-invariant direct methods	Extinction coefficient: 0.0137 (8)

### Special details

**Table:**

Geometry. All e.s.d.'s (except the e.s.d. in the dihedral angle between two l.s. planes) are estimated using the full covariance matrix. The cell e.s.d.'s are taken into account individually in the estimation of e.s.d.'s in distances, angles and torsion angles; correlations between e.s.d.'s in cell parameters are only used when they are defined by crystal symmetry. An approximate (isotropic) treatment of cell e.s.d.'s is used for estimating e.s.d.'s involving l.s. planes.
Refinement. Refinement of *F*^2^ against ALL reflections. The weighted *R*-factor *wR* and goodness of fit *S* are based on *F*^2^, conventional *R*-factors *R* are based on *F*, with *F* set to zero for negative *F*^2^. The threshold expression of *F*^2^ > σ(*F*^2^) is used only for calculating *R*-factors(gt) *etc*. and is not relevant to the choice of reflections for refinement. *R*-factors based on *F*^2^ are statistically about twice as large as those based on *F*, and *R*- factors based on ALL data will be even larger.

### Fractional atomic coordinates and isotropic or equivalent isotropic displacement parameters (Å^2^)

**Table:**

	*x*	*y*	*z*	*U*_iso_*/*U*_eq_	
C1	0.2297 (3)	0.0215 (7)	1.03419 (17)	0.0397 (7)	
C2	0.3291 (3)	−0.0895 (7)	1.08624 (18)	0.0425 (8)	
C3	0.3353 (4)	−0.0151 (10)	1.15353 (19)	0.0551 (9)	
H3	0.4019	−0.0941	1.1881	0.066*	
C4	0.2433 (5)	0.1749 (10)	1.1691 (2)	0.0629 (11)	
H4	0.2482	0.2279	1.2143	0.075*	
C5	0.1426 (5)	0.2888 (9)	1.1178 (2)	0.0591 (10)	
H5	0.0795	0.4170	1.1285	0.071*	
C6	0.1361 (4)	0.2117 (9)	1.0508 (2)	0.0501 (9)	
H6	0.0683	0.2881	1.0165	0.060*	
C7	0.2077 (4)	0.1136 (7)	0.91258 (19)	0.0446 (8)	
C8	0.2212 (5)	−0.0244 (9)	0.84652 (19)	0.0564 (10)	
H8A	0.2136	−0.2284	0.8507	0.068*	
H8B	0.3111	0.0170	0.8378	0.068*	
N1	0.2235 (3)	−0.0623 (6)	0.96571 (15)	0.0421 (6)	
H1N	0.234 (4)	−0.233 (5)	0.957 (2)	0.050*	
O1	0.1903 (4)	0.3694 (5)	0.91641 (16)	0.0658 (8)	
Cl1	0.44859 (10)	−0.3266 (2)	1.06732 (5)	0.0583 (3)	
Br1	0.08344 (5)	0.10591 (13)	0.77113 (2)	0.0742 (3)	

### Atomic displacement parameters (Å^2^)

**Table:**

	*U*^11^	*U*^22^	*U*^33^	*U*^12^	*U*^13^	*U*^23^
C1	0.0435 (16)	0.0345 (16)	0.0401 (17)	−0.0053 (14)	0.0065 (13)	−0.0034 (14)
C2	0.0444 (17)	0.0402 (17)	0.0426 (18)	−0.0046 (14)	0.0083 (14)	−0.0022 (14)
C3	0.067 (2)	0.057 (2)	0.0396 (19)	−0.0064 (19)	0.0055 (17)	−0.0013 (18)
C4	0.080 (3)	0.067 (3)	0.047 (2)	−0.010 (2)	0.025 (2)	−0.013 (2)
C5	0.065 (2)	0.055 (2)	0.064 (2)	0.002 (2)	0.029 (2)	−0.013 (2)
C6	0.0485 (19)	0.048 (2)	0.054 (2)	0.0036 (16)	0.0099 (16)	−0.0042 (18)
C7	0.0530 (19)	0.0367 (18)	0.0393 (18)	0.0005 (14)	−0.0010 (14)	−0.0023 (14)
C8	0.076 (3)	0.051 (2)	0.0387 (19)	0.009 (2)	0.0048 (17)	−0.0008 (18)
N1	0.0542 (16)	0.0332 (14)	0.0353 (15)	0.0038 (13)	0.0016 (12)	−0.0039 (12)
O1	0.108 (2)	0.0331 (14)	0.0523 (17)	0.0082 (14)	0.0087 (16)	−0.0002 (11)
Cl1	0.0509 (5)	0.0628 (6)	0.0573 (6)	0.0146 (4)	0.0025 (4)	−0.0032 (5)
Br1	0.0792 (4)	0.0936 (5)	0.0427 (3)	−0.0058 (3)	−0.0035 (2)	0.0111 (2)

### Geometric parameters (Å, °)

**Table:**

C1—C2	1.381 (5)	C5—H5	0.9300
C1—C6	1.385 (5)	C6—H6	0.9300
C1—N1	1.416 (4)	C7—O1	1.223 (4)
C2—C3	1.381 (5)	C7—N1	1.332 (5)
C2—Cl1	1.734 (4)	C7—C8	1.506 (5)
C3—C4	1.365 (6)	C8—Br1	1.916 (4)
C3—H3	0.9300	C8—H8A	0.9700
C4—C5	1.385 (7)	C8—H8B	0.9700
C4—H4	0.9300	N1—H1N	0.839 (19)
C5—C6	1.377 (6)		
			
C2—C1—C6	118.5 (3)	C5—C6—C1	120.6 (4)
C2—C1—N1	120.2 (3)	C5—C6—H6	119.7
C6—C1—N1	121.3 (3)	C1—C6—H6	119.7
C1—C2—C3	121.2 (3)	O1—C7—N1	124.0 (4)
C1—C2—Cl1	119.8 (3)	O1—C7—C8	121.4 (4)
C3—C2—Cl1	119.1 (3)	N1—C7—C8	114.5 (3)
C4—C3—C2	119.7 (4)	C7—C8—Br1	111.8 (3)
C4—C3—H3	120.1	C7—C8—H8A	109.3
C2—C3—H3	120.1	Br1—C8—H8A	109.3
C3—C4—C5	120.1 (4)	C7—C8—H8B	109.3
C3—C4—H4	119.9	Br1—C8—H8B	109.3
C5—C4—H4	119.9	H8A—C8—H8B	107.9
C6—C5—C4	119.8 (4)	C7—N1—C1	125.0 (3)
C6—C5—H5	120.1	C7—N1—H1N	115 (3)
C4—C5—H5	120.1	C1—N1—H1N	120 (3)
			
C6—C1—C2—C3	0.4 (5)	C2—C1—C6—C5	0.3 (6)
N1—C1—C2—C3	−178.7 (3)	N1—C1—C6—C5	179.3 (4)
C6—C1—C2—Cl1	−179.6 (3)	O1—C7—C8—Br1	−46.2 (5)
N1—C1—C2—Cl1	1.3 (4)	N1—C7—C8—Br1	137.2 (3)
C1—C2—C3—C4	−1.1 (6)	O1—C7—N1—C1	−3.4 (6)
Cl1—C2—C3—C4	178.9 (3)	C8—C7—N1—C1	173.1 (3)
C2—C3—C4—C5	1.1 (7)	C2—C1—N1—C7	−134.5 (4)
C3—C4—C5—C6	−0.5 (7)	C6—C1—N1—C7	46.5 (5)
C4—C5—C6—C1	−0.2 (6)		

### Hydrogen-bond geometry (Å, °)

**Table:**

*D*—H···*A*	*D*—H	H···*A*	*D*···*A*	*D*—H···*A*
N1—H1N···O1^i^	0.84 (2)	2.05 (2)	2.852 (4)	160 (4)
C8—H8B···Cl1^ii^	0.97	3.09	3.765 (5)	128

Symmetry codes: (i) *x*, *y*−1, *z*; (ii) −*x*+1, −*y*, −*z*+2.
